# Complex mechanics of the heterogeneous extracellular matrix in cancer

**DOI:** 10.1016/j.eml.2018.02.003

**Published:** 2018-05

**Authors:** Andrea Malandrino, Michael Mak, Roger D. Kamm, Emad Moeendarbary

**Affiliations:** a Institute for Bioengineering of Catalonia, Barcelona, Spain; b European Molecular Biology Laboratory, Barcelona, Spain; c Department of Biomedical Engineering, Yale University, New Haven, CT, USA; d Departments of Biological Engineering and Mechanical Engineering, Massachusetts Institute of Technology, Cambridge, MA, USA; e Department of Mechanical Engineering, University College London, London, UK

## Abstract

The extracellular matrix (ECM) performs many critical functions, one of which is to provide structural and mechanical integrity, and many of the constituent proteins have clear mechanical roles. The composition and structural characteristics of the ECM are widely variable among different tissues, suiting diverse functional needs. In diseased tissues, particularly solid tumors, the ECM is complex and influences disease progression. Cancer and stromal cells can significantly influence the matrix composition and structure and thus the mechanical properties of the tumor microenvironment (TME). In this review, we describe the interactions that give rise to the structural heterogeneity of the ECM and present the techniques that are widely employed to measure ECM properties and remodeling dynamics. Furthermore, we review the tools for measuring the distinct nature of cell–ECM interactions within the TME.

## 1 General overview

We review the specific roles of ECM mechanics in tumor progression, with emphasis on mechanobiological phenomena arising from the complex interactions between the heterogeneous ECM microenvironment and tumor and stromal cells. While complementing other reviews (see [[1], [2], [3], [4], [5], [6], [7], [8], [9]]) we highlight (i) the heterogeneity of ECM mechanical properties as a result of dynamic cellular interactions and remodeling processes and (ii) the current advances in measuring these properties. Here we focus on solid tumors, in which the ECM, acting as a scaffolding medium, has proven to impact cell mechanical responses, such as migration, contractile forces, mechanotransduction, and mechanosensing, which in turn influence the degree of tumor malignancy and metastatic potential. In the following sections, we cover ECM functions, cell–ECM interactions, and advances in biophysical techniques for corresponding measurements.

## 2 The ECM

Tissues are typically comprised of ECM, cells, blood-filled vascular space, in addition to a collection of other proteins used for signaling between cells, but the proportions differ drastically among anatomical locations. Some, such as cartilage or the cornea, show low cellularity (and lack a vascular supply), so are primarily comprised of ECM, having unique mechanical, and in the case of the cornea, optical properties. Others, such as the heart or pancreas, for example, are dominated by their cellular content, both in terms of their function and their mechanical stiffness. In tissues, structure generally follows function [10].

The ECM is comprised of approximately 300 proteins, and they serve a variety of functions. Some cross-link to form into long filaments that in turn bundle into fibers and serve largely a structural role: collagen, elastin and fibronectin are common [1]. But even at this level, there are fundamental mechanical differences—e.g., elastin exhibits linear, entropic elastic behavior and can sustain high levels of strain without fracture, whereas collagen is highly non-linear, much stiffer, and strains very little before fracture [[11], [12]]. As with most filaments, both collagen and elastin tend to be stiff under tension, but buckle under compressive stress. Other constituents serve different functions, such as the proteoglycans (PGs), which are glycoproteins decorated with highly charged, space-filling glycosaminoglycans (GAGs). Due to their high negative charge density, they primarily resist compressive stress, and are especially important in cartilaginous tissues [13].

Fiber arrangement can also be an important determinant of ECM material properties. Collagen and elastin in particular can align into cylindrical chords such as tendons and ligaments, or sheet-like structures, stiff in the plane of the sheet, but compliant perpendicular to it [[14], [15], [16]]. Tissues like the cornea or the intervertebral disk, are especially interesting examples in which the collagen is arranged in layers, alternating in fiber orientation [[13], [17]]. Non-linearity can arise from a variety of sources, but in collagen-rich tissues, it often results from a progressively increasing fraction of the filaments becoming taut with a concomitant increase in stiffness as the tissue is strained [[12], [18]].

One of the unique features of biological tissues that help to distinguish them from abiotic ones, is their ability to remodel in response to various factors, an effect largely mediated by the cellular content and the ability of the cells to sense and respond to mechanical stimuli. Cells can alter the ECM by synthesizing new matrix proteins, altering the extent of crosslinking, or secreting enzymes that selectively break down matrix elements [10]. Cross-linking occurs via several mechanisms, but disulfide bonding is common, occurring in many collagens and laminins. Matrix degradation is mediated again by a variety of proteins, including matrix metalloproteases (MMPs), ADAMTS proteases, elastaces, and cathepsins [[19], [20]]. Most have specific sequence targets enabling the cells to fine-tune the mechanical properties of their environment. In order to respond to stress, the cells need to sense it, and this is done via several families of cell–matrix adhesion molecules, but predominantly proteins in the integrin family. These are prominent transmembrane proteins that link the ECM to the intracellular structural members such as the actin cytoskeleton [21], and are again, highly selective in terms of their specific binding partners. For example, laminin binds to dimers consisting of the beta-3 integrin coupled with alpha 3, 6, or 7, and is largely responsible for tethering the basement membrane associated with blood vessels to the vascular endothelium [[22], [23]]. Finally, although they do not participate in the load-bearing function of tissues, the ECM contains a plethora of signaling molecules, and these often bind to specific ECM proteins. Thus, the ECM serves as an effective reservoir of factors that can, in turn, regulate numerous cell functions, such as growth, migration, and protein synthesis and secretion.

As the architecture of the ECM, composed of many constituents linked together into a complex network, can contribute to functional roles and guide cell behavior, various imaging methods have been applied to directly visualize local and global organizational patterns. Electron microscopy provides high resolution imaging of individual fibers, revealing their fine structures [24]. To image ECMs along with cells under various physiologically relevant conditions, optical imaging enables non-destructive visualization, which can also be performed with live cells. Common methods for optical imaging of the ECM, particularly for common matrix proteins collagen I and fibrin, include fluorescence excitation and confocal reflectance [[25], [26], [27]]. For collagen I, second harmonic generation microscopy is also applicable and has been used to visualize collagen in tissues with various diseases, including cancer, fibrosis, and atherosclerosis [28]. Imaging studies have shown that many common ECMs are organized into a complex network of interconnected fibers. In stromal tissues, cells are typically encapsulated inside this 3-dimensional fiber matrix, which provides a physically and geometrically distinct environment compared to traditional cell culture conditions on flat (2D) substrates [29].

Imaging of the ECM in cancer specimens, from preclinical and clinical biopsies and in vitro samples, demonstrate distinctive features, including increased collagen density and matrix alignment in the vicinity of tumors [[30], [31]]. These ECM signatures are correlated with disease progression and poor prognosis [30]. Tumor tissue environments, particularly from aggressive tumors, have also been shown to be stiffer [[30], [32]], potentially due to a combination of increased ECM concentration, higher matrix crosslinking, and nonlinear stiffening of the ECM fiber network under cancer cell generated tension [[33], [34], [35]], discussed more later.

## 3 Modes of interaction between cells and the ECM

### 3.1 Local ECM tension, degradation, and production

Through integrins, cells engage the ECM utilizing cytoskeletal contractile forces generated by molecular motors (myosins) walking on actin filaments. Contractile forces are transmitted to the ECM network, leading to matrix stiffening [35]. Stiffer substrates induce increased cell-generated tension [[36], [37]], generating a positive mechanical feedback. In addition to pulling on matrix fibers, cells can synthesize and degrade the ECM through different types of MMPs specialized in degrading different ECM proteins. MMP-1, for instance, cleaves fibrillar collagen I, while MMP-2 and MMP-9 degrades the basement membrane [38], consisting largely of collagen IV and non-collagenous components such as laminin [[2], [39]]. Production of new ECM occurs as a highly integrated process in which ECM molecular components are synthesized and packaged inside the cell, secreted, and self-assemble into the existing matrix [2]. Depending on the microenvironment, highly ordered fiber networks (e.g. in some connective tissues) or amorphous gels (e.g. in the brain) can be generated. The molecular content and network architecture of the ECM determine mechanical and functional properties and cell–matrix interactions [[3], [4], [40], [41], [42], [43], [44]].

### 3.2 Cell motility and ECM

**Fig. 1:**
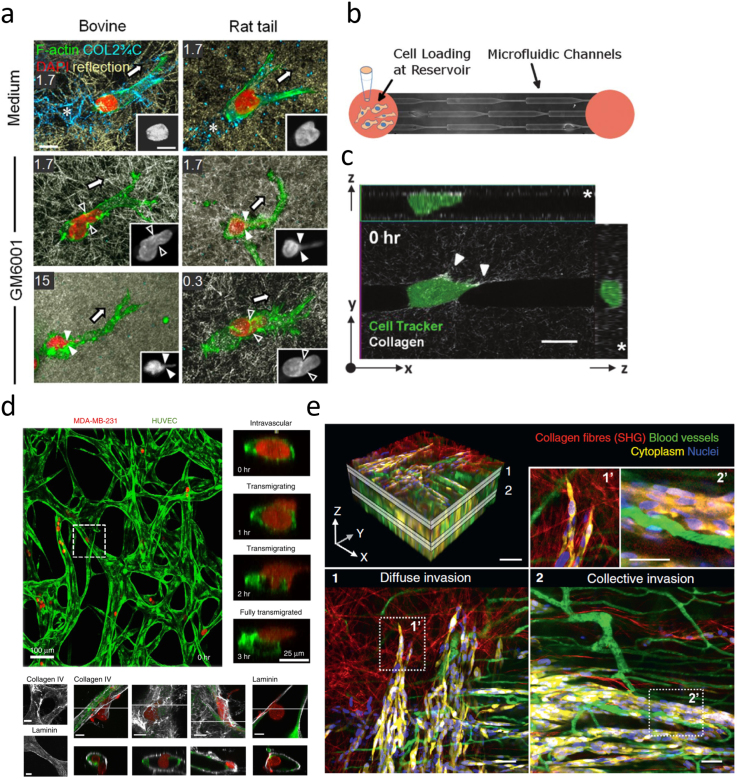
Capturing the interactions between cancer cells and their microenvironment at varying levels of complexity. (a) A cancer cell navigates through a reconstituted 3D collagen ECM with or without inhibition of MMPs via GM6001 [45]. (b,c) Microfluidic (b) and micropatterning (c) techniques can reproduce and isolate key features of the TME, such as confinement or ECM tracks [[47], [48]]. (d) In vitro co-culture systems can capture specific cell–cell and cell–ECM interactions, such as a cancer cell extravasating from a microvascular network (top) or breaching the basement membrane during extravasation (bottom) [49]. (e) Intravital imaging in mice captures complex TMEs with multiple cell types and in vivo ECMs [50].

The surrounding ECM influences cell behavior, including growth and migration. Collagen density determines the pore size of a collagen matrix. Reduced pore sizes, especially when smaller than the cell nucleus, restrict the mechanical motion of cells, requiring motile cells to undergo substantial deformations and utilize MMPs [[45], [46]]. Inhibition of MMPs reduces cell migration rates in dense matrices but less so in sparse matrices [45] (Fig. 1 a). MMPs therefore may be expendable in sparse matrices.

Although ECMs are often quantified by bulk metrics such as average pore size or average fiber length, they have a high degree of heterogeneity due to the intrinsic disorder of the fiber network. Micro-patterning and manipulation methods have been applied to accentuate certain local features and to determine their impact on cell behavior. Microchannels of variable dimensions and bifurcating paths revealed that migratory decision making depends on both the dimensionality and directionality of the path [51]. Aligned paths with larger dimensions are favored. Paths with cross-sections smaller than the cell nucleus require additional time for cell transmigration as the nucleus deforms under persistent force generation [[47], [52]] (Fig. 1 b). Aligning collagen matrices by controlling flow and temperature during gelation leads to cells that preferentially extend and migrate in the same direction [[41], [53]]. Cells themselves can also induce ECM alignment by applying tension [[54], [55]]. Furthermore, cells have been shown to migrate along gradients of substrate stiffness and ligand density [[56], [57], [58]]. Cell migratory patterns are therefore biased by local ECM properties. Importantly, these features (dimensionality, alignment, stiffness) are all inducible by cells themselves via force generation, matrix degradation and synthesis, and secretion of crosslinking factors.

In TMEs, enhanced force generation by aggressive cells [37], along with secretion of collagen crosslinking factors [33], can lead to local stiffening of the matrix and alignment of matrix fibers, generating features conducive toward invasion. Additionally, MMP activity by tumor cells can generate cell-scaled tracks along migratory paths [59]. Tumors (and stromal cells) therefore act as local sources of ECM remodeling, resulting in heterogeneous spatial and temporal profiles of the ECM network. These profiles can then influence the migration of surrounding cells. In micropatterned collagen tracks that mimic tube-like paths cleared by MMP-mediated degradation, cancer cells have been shown to migrate with increased speeds in an MMP-independent manner [48] (Fig. 1 c), as cells in these paths do not need to squeeze through or clear constrictive mechanical barriers.

### 3.3 Cell populations and ECM

The tumor stroma encapsulates many other cell types in addition to the cancer cells. Fibroblasts, immune cells, and endothelial cells have all been shown to interact with cancer cells and influence invasion and metastasis. Macrophages, which secrete TNFα and TGFβ, stimulate MMT1-MMP and MMP1 in cancer cells, leading to increased migratory speed and persistence in collagen matrices [60]. Cancer-associated fibroblasts (CAFs) influence the TME in a number of ways. They can use transmembrane proteins to pull on cancer cells and lead them to disseminate away from the local bulk tumor and into the ECM [61]. This may facilitate the invasion of tumors that tend to stay localized. CAFs also release pro-inflammatory factors, which promote the recruitment of macrophages, MMP activity, and angiogenesis [62]. To spread to distant sites, cancer cells need to transmigrate across endothelial barriers in order to access and exit from the vascular system. Transmigration involves both squeezing through endothelial junctions and penetrating through the basement membrane, a relatively thin matrix produced by endothelial cells that separate the endothelium from the surrounding connective tissue (Fig. 1 d). Different MMPs are required to degrade the basement membrane and interstitial matrices. Additionally, integrin β1 appears to be critical for tumor cells to penetrate through the basement membrane after migrating through endothelial junctions, as cells with integrin β1 knocked down appear to be able to transverse endothelial junctions but not the basement membrane [[23], [49]].

The TME therefore hosts many diverse mechanical and biochemical interactions during cancer progression and metastasis. Some of these interactions are being targeted actively in therapeutic development, such as angiogenesis, MMPs, and chronic inflammation [[5], [63]], whereas other factors such as mechanical interactions and force generation, which are also important in normal tissue maintenance and function, may require novel strategies. Various methods, from 3D mono- and co-culture systems to micropatterning and microfluidics to in vivo imaging [50] (Fig. 1 e), can address the different degrees of complexity between cells and their surrounding environment (Fig. 1).

## 4 Measuring ECM physical properties in cancer

**Fig. 2:**
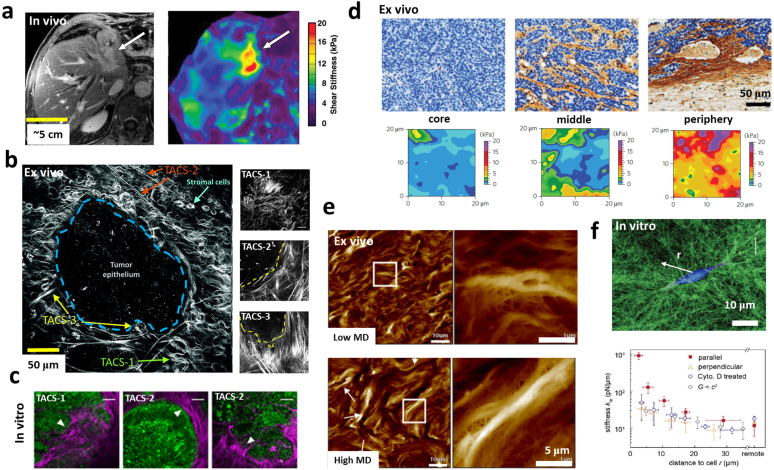
Characterization of physical properties of tumor tissue and associated ECM via different tools. (a) Significant stiffening of the tumor (from an average of 4.3 kPa in liver parenchyma to an average of 15.5 kPa at the tumor site) is probed via magnetic resonance elastography (right image) of cholangiocarcinoma invaded surrounding left lobe of human liver (arrow in the left T1-weighted magnetic resonance image) [70] (b) Second harmonic generation image of an ex vivo mouse mammary tumor indicates three tumor associated collagen signatures (TACS): The first signature (TACS-1) is related to a wavy collagen similar to a normal mammary gland but with increased density at regions near tumor. The second (TACS-2) and third (TACS-3) signatures can be characterized by straightened and aligned collagen fibers oriented parallel or perpendicular to the tumor edge respectively [74]. A clearer representation of different TACS is shown in the right panels [31]. (c) In vitro model of CT26 tumor spheroid embedded in 3D collagen I exhibited similar TACS [75]. (d) The stiffness maps (bottom panels) of mouse mammary tumors extracted via AFM indentations show extreme tissue stiffening (∼ 5-fold) in peripheral regions compared to the tumor core which can be correlated to significant changes in collagen density, structure, and morphology as well as cell density as indicated in immunohistochemistry images in top panels [76]. (e) Topographic maps of human breast tissue measured via AFM show more bundled and aligned collagen fibers in patients with high mammographic density (MD) compared to patients with low MD [77]. (f) Quantifying mechanics of collagen I under the influence of single cancer cells. Confocal reflectance microscopy (top panel) shows remodeling of the collagen network around an MDA-MB-231 breast cancer cell. Quantification of the network stiffness via optical tweezers indicates stiffening of the collagen network at long distances (∼>20 μm) away from the cancer cell [78].

Abnormal ECM composition, architecture and stiffness have been identified to play integral roles in cancer progression at all steps of metastasis [[6], [40]]. It is crucial to measure and quantify the changes in ECM properties since they regulate tumor growth, transformation to malignancy, and invasion [7]. Depending on in vivo, ex vivo, and in vitro conditions and the associated technological limitations, tumor tissues and cancer associated ECMs have been mechanically characterized from macro to micro and nano scales. Elastography techniques based on **ultrasonography** [[64], [65]], **optical coherence tomography** [[66], [67], [68]], and **magnetic resonance imaging** [[69], [70]] (Fig. 2 a) are among the widely-employed techniques for non-invasive measurement of in vivo mechanical properties of tumors in patients and animals. Elastography measurements revealed significant stiffening of tumor tissues in vivo, particularly for malignant tumors, compared to normal tissues [[71], [72]]. While these in vivo mechanical measurements can identify the presence of abnormal changes in the stiffness of the tumor bulk and can be considered as a diagnostic approach alternative to conventional palpation methods, they lack the resolution to dissect the contribution of various tissue elements, such as cells and ECMs, and the role of intra-tumor stresses. Fundamentally, the increased stiffness of tumors in vivo can result from the combined effects from the alterations in cellular and extracellular compositions and structures, such as excessive proliferation of cancer cells, causing ECM remodeling and a build-up of growth induced solid stress within the tumor [73], and changes in stromal cells and vascular architecture, causing unnatural interaction of blood flow within and surrounding the tumor and build-up of interstitial fluid pressure [8]. A myriad of other techniques, mostly based on ex vivo and in vitro conditions, have been employed to provide high resolution physical characterization of the local tumor microenvironment at microscales and the capacity to dissect the contribution of cells and ECM physical properties, discussed below and in Fig. 2.

### 4.1 Optics-based techniques

Optical techniques have been widely applied to quantify changes in ECM composition and remodeling in ex vivo slices of tumors or in vitro assays. **Confocal microscopy** in reflectance or fluorescence modes has been applied to reveal the images of ECM structures mainly in thin tissue slices, due to limited optical penetration depth [79]. While **reflectance confocal microscopy** is the most straightforward label-free method of characterizing the remodeling of pre-existing ECM in simple in vitro assays, immunofluorescence in combination with **fluorescence confocal microscopy** provides the ability to probe remodeling and deposition of multiple types of tumor associated ECMs, particularly in ex vivo tumor slices, with submicron resolution [79]. Providing high penetration depth (up to 1 mm) and contrast in addition to submicron resolution, **multiphoton microscopy** has been an extremely useful optical tool for capturing high resolution images of ECM alignment particularly in live tumor specimens [[80], [81]]. By taking advantage of the large penetration depth and high sensitivity of **second harmonic generation** (SHG) for label-free imaging of collagen structures (Fig. 2 b–c), it has been possible to perform live imaging of the organization of the collagen matrix and its interactions with other fluorescently labeled ECM proteins, cancer cells, and stromal cells [[74], [82], [83]]. Another set of emerging optical tools involve the extraction of mechanical properties based on unique interactions between photons and phonons and the changes in the behavior of optically generated acoustic waves upon experiencing different material properties [79]; **Brillouin microscopy** is an opto-mechanical characterization method that has been recently integrated with confocal microscopy, allowing non-contact extraction of high resolution stiffness maps of biological samples [[84], [85]] and potentially tumor tissues [86].

### 4.2 Mechanics-based measurements

Since direct application of forces typically requires the contact between a mechanical probe and the sample, mechanics based techniques to measure tumor stiffness are mostly performed in ex vivo or in vitro conditions. Conventional engineering methods such as compression and shear tests have been applied to quantify the stiffness of ex vivo tumors [[71], [87]]. However, these bulk measurements do not have sufficient accuracy and sensitivity to capture local heterogeneous mechanical properties of tumors. Indentation is a robust mechanical characterization method of soft materials. In particular, by tuning the size of the indenter and the sensitivity of the mechanical apparatus, indentation can offer high resolution micro/nano scale quantification [[88], [89]].

Indentation and topography tests via **atomic force microscopy**, a very high resolution versatile tool for studying biological samples, have been pivotal in the field of cancer biomechanics and revealed a myriad of mechanical information about the TME at molecular, cellular, and tissue levels [[90], [91]]. Nanomechanical indentation tests and topography measurements, performed via AFM on ex vivo tissue slices, revealed a high degree of heterogeneity in the stiffness and collagen architecture of tumors [92]. Interestingly, at the core of a tumor, where cancer cells are abundant, the tumor exhibits a soft mechanical signature while the adjacent peripheral regions, where collagen alignment is apparent, are stiffened [[76], [93]] (Fig. 2 d). Based on recent AFM measurements, it has been suggested that the remodeling of ECM microarchitecture, particularly in collagen, leads to tumor stroma stiffening which triggers the epithelial to mesenchymal transition, invasion of tumor cells, and metastasis [[77], [94], [95]] (Fig. 2 e). Moreover, it has been concluded that in addition to the ECM, the tumor epithelium and the tumor-associated vasculature contribute to the stiffening of the tumor stroma, as quantified via AFM [96].

In addition to quantifying physical properties of tumors in vivo and ex vivo conditions, numerous in vitro assays have been used to study the effects of cancer cells and tumor associated stromal cells, such as fibroblasts, on 3D remodeling of naturally derived ECMs [97]. Interestingly, mechanical quantification via AFM of gels embedded with fibroblasts, revealed that activation of YAP mechano-signaling in cancer-associated fibroblasts induces extreme ECM remodeling and stiffening, more than 8-fold compared to stiffening by normal fibroblasts, which contributes to the tumor bulk stiffening observed in vivo [98]. **Magnetic and optical tweezers** are also among promising high resolution mechanical techniques that have been recently employed to characterize ECM properties [[99], [100], [101]]. Interestingly, measurements of cancer cell-induced ECM contractions at the single cell level using optical tweezers revealed long-ranged stress stiffening of the ECM correlated with ECM remodeling and non-linear elasticity as well as inelastic behavior of collagen networks [[78], [102]] (Fig. 2 f).

## 5 Techniques for measuring cell–ECM interactions

**Fig. 3:**
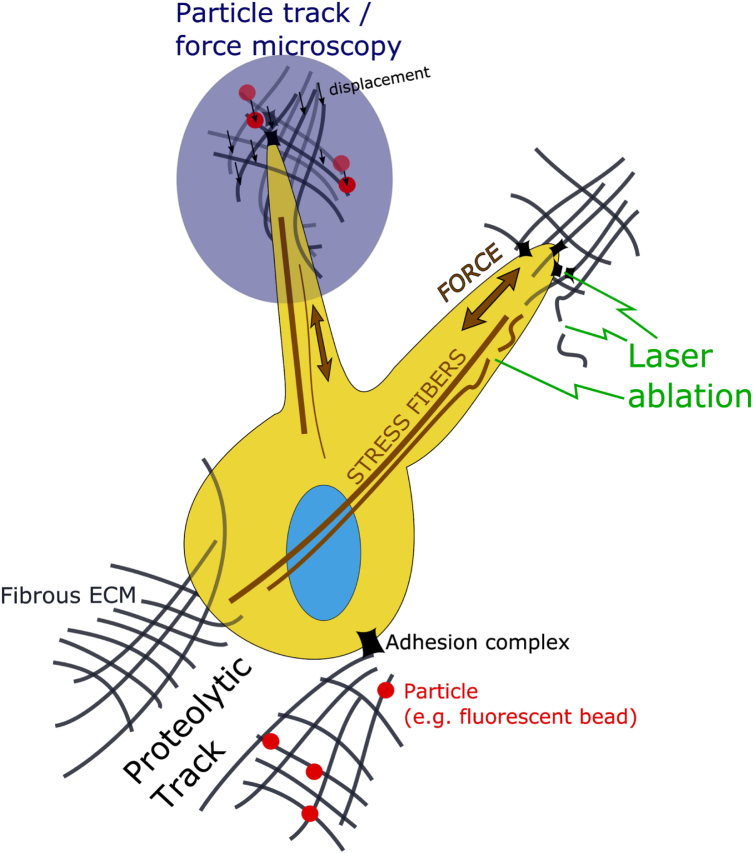
Schematic of cell–ECM interactions that can be measured in 3D by utilizing the reviewed methodologies. Some examples are illustrated for (i) measuring ECM displacements resulting from contractile forces from tracking of fluorescent particles or cross-correlations of ECM images before and after cellular force application (ii) measuring ECM or stress fiber relaxation after laser ablation and (iii) imaging and assessing cellular proteolytic processes in the ECM.

To measure the interactions between cells and their surrounding matrices, probes and methodologies are required at the cellular and subcellular resolutions (Fig. 3) and over biologically relevant time scales. The readouts of interest of many advanced techniques, which we review here, are often forces and matrix architecture.

### 5.1 3D traction force techniques

The techniques that back-calculate forces that cells apply on planar substrates – mainly as a 2D geometrical problem – have seen rapid development and adoption and reached good accuracy (see for instance [103]). Most of these methods use elastic substrates, such as polyacrylamide gels with embedded tracer particles, and computational algorithms to extract cell-generated forces based on substrate deformations, captured in microscopy images of tracer displacement profiles. Also, for such force inference, micropatterned substrates (*e.g.* micropillars of known stiffness) that can be elastically deformed and followed over time have proved useful and accurate to measure forces [[104], [105]]. These methods have been extended to cells surrounded by a 3D ECM and have shown presumed feasibility when the ECM itself can be treated as an elastic continuum whose stiffness is used for back-calculation of forces from the tracer bead displacement data [[106], [107], [108]]. However, when cells alter this stiffness spatiotemporally, many 3D traction force microscopy techniques lose accuracy [109]. Stiffness alteration by cell activity includes local proteolytic degradation, inelastic remodeling (such as matrix densification), and any non-linearity of stiffness values.

Algorithms for 3D determination of displacement fields from the tracking of fluorescent beads or cross-correlation of 3D volumetric image data must ensure that the resulting distribution of all forces applied from cell processes (such as filopodial dynamics) are mechanically self-equilibrated. This is often accomplished with inverse optimization procedures and can be a non-trivial task when multicellular entities are under examination. Powerful algorithms have been recently developed for 3D traction measurements from fluorescence microscopy [[108], [109], [110]]. When combined with experimental techniques such as matrix fluorescent labeling or reflectance imaging (e.g. collagen or fibrin gel fibers, [[110], [111]]), these algorithms have provided reliable results at the subcellular resolution.

### 5.2 Particle tracking methods

In 3D traction microscopy of cells in fibrous biopolymer ECMs, fiber bundles or exogenous beads covalently attached to the fiber lattice can be spatiotemporally tracked. Generalizing this concept, single molecules or macromolecular assemblies are also particles that can be tracked using computational approaches. Particle tracking has thus vast applicability. It has been used for the study of the dynamics of cytoskeletal microtubules ends, showing how alternating periods of growth and shrinkage modulate cell architecture and cytoskeletal forces [112]. Particle tracking is suitable to extract relevant data from highly dynamical processes, such as the assembly and disassembly of focal adhesion complexes. In one study of this kind, several features – e.g. geometry, fluorescence intensity, and position – of Paxillin and FAK were tracked with available tools and have helped determine adhesion lifetimes and turnover rates [113].

Moreover, by using video microscopy and recording the time-dependence of average quantities such as the mean square displacement of moving particles, tracking can inform on the modes of motion of molecular entities. In turn, these modes relate to diffusion processes [114]. For instance, membrane dynamics studies could detect both Brownian and non-Brownian motions and transition phases among modes of motions, revealing spatiotemporal phenomena such as the partition of molecules into different subspecies or the transition to active motion modes, such as the binding to a motor protein [[115], [116]]. Also, passive microrheology – measuring rheological properties from the Brownian motion of ECM-embedded particles – can provide important data to understand the dynamics of cell–ECM interaction. In a recent paper, Schultz and coworkers functionalized a cell-laden hydrogel and tracked microenvironmental changes at multiple time and size scales. The authors could correlate cell-mediated initial proteolytic changes in the ECM farther away from the cells to cytoskeletal tension across the material. On longer timescales, particle tracking provided evidence for a transition of the pericellular ECM-mimicking hydrogel from an elastic gel to a viscous liquid, mediated by degradation processes [117].

### 5.3 Laser ablation

To probe mechanical stresses at the subcellular level one can artificially relax the tension built by a molecular assembly. The laser ablation technique can be used for this purpose. The technique consists of sublimating or evaporating a portion of the molecular assembly using the energy of a focused laser beam. Laser ablation acts as a nano scale scissor that results in expansion (or shrinkage) of tissues, revealing the tension (or compression) that kept the tissue together before ablation. Laser ablation can be used in combination with bead tracking and/or knowledge of material properties: the tracking of movements of the surrounding ECM when laser ablating acto-myosin assemblies is used to back-calculate the mechanical stress. Laser ablation studies have elucidated the dynamics of multicellular cooperation mediated by the ECM, resulting in a rapid force transmission to the ECM when single stress fibers are disrupted [[118], [119]]. Viscoelastic effects must be expected in cell mechanics applications. Therefore ablation and related time-scales of the expansions/shrinkages can reveal both viscous and elastic constants [[118], [120]]. Laser ablation shares with all of the cell–ECM tools introduced so far an important limitation that concerns the proteolytic- and/or remodeling-driven stiffness modifications, which undermine the accuracy of the back-calculated values of tension. Yet, laser ablation is a powerful technique for selectively probing cell–ECM-related structures such as cytoskeletal cables, cell–matrix adhesion proteins, and ECM components.

### 5.4 Analysis of proteolytic tracks

Cell migration, especially during invasive spreading through fibrous 3D ECMs, often entails proteolytic activity for fiber breakdown. Proteolytic remodeling, via upregulation of MMPs, serine and cysteine proteases, results in cells forming tracks in the ECM. This proteolytic cleavage occurs at the subcellular, cell–ECM interface, and is often co-localized with the cell adhesion molecular machinery [121]. Imaging of the ECM structure (through confocal reflectance or fluorescence microscopy) is often used to study these processes. Also, fluorophore dequenching highlighting proteolytic activity, combined with analyses of cell deformation and migration speed has delivered relevant insights. With these combined tools, proteolytic activity can be measured on cancer cells seeded within 3D collagen lattices containing labeled type I collagen monomers [[122], [123]]. Furthermore, several techniques for in vivo research have been developed to image the effect of MMPs in TMEs, such as optical imaging, positron emission tomography, single photon emission computed tomography, and magnetic resonance imaging. These target cancer progression-mediated ECM changes through the use of contrast agents linked to MMP inhibitors or to engineered substrates amplified during enzymatic processes [124].

### 5.5 Examples of cell–ECM interaction dynamics distinct in cancer

The techniques covered so far have been applied to study the mechanical interactions between the cell types and the complex landscape of ECM proteins characterizing the TME. As for cell-generated forces, these techniques have reported differences between non-cancerous and cancerous cells. For instance, traction force microscopy has measured larger contractile forces generated by more malignant cells on different substrates, compared to non-metastatic cells [37]. Another example related to mechanical signaling is the possibility of studying integrins as mechanoreceptors and their distinct features when interacting with the TME. Based on traction force measurements, it has been shown that the ability to exert Rho GTPase-dependent cytoskeletal tensions is functionally linked to the ECM stiffness. This has provided evidence of an important mechanism by which cells may use ligands to feel the crosslinking of exogenous ECMs [125].

Although mostly focused on cells seeded on 2D substrates, laser ablation has also been used to study force propagation in the ECM. Recently, laser ablation was used to measure the mechanical tension within a collagen gel 48h after seeding of cancer spheroids [75]. It was shown that contractile forces rapidly deform the surrounding ECM in a centripetal fashion. Interestingly, selectively ablating the 3D collagen lattice reduced spheroid cell spreading, suggesting a prestress-dependent mechanotransduction regulation of cancer invasion.

Finally, particle tracking of intracellular beads has been used to show further linking of intracellular regulation and stiffening to cell motility and perturbed mechanotransduction in breast cancer, which further confirms that the adaptation of intracellular contractility and stiffness are ECM stiffness-dependent [126]. Matrix-embedded beads can also be tracked in combination with proteolytic tracks analyses [127]. In this study, beads were tethered to collagen I fibers near migrating fibrosarcoma cells in the absence and presence of proteolytic inhibitors and acto-myosin contractile forces. Taking the axis of cell migration as reference during forward cell motion, ECM release due to proteolytic activity near the trailing edge was measured. ECM degradation was asymmetric to the axis and produced inelastic deformation, while symmetry was observed at the ECM deformations near the leading edge, with these deformations being elastically recoverable.

Beyond the reductionist approach employed in many tumor biology studies, one important effort would be to channel these methodologies to study mechanical signaling when multiple cell types and macromolecular assemblies that characterize cancer complexity are integrated [[19], [128]]. Many cell type-specific processes contribute toward furbishing the TME, such as those of immune and endothelial cells, mesenchymal stem cells, as well as pericytes and cancer-associated fibroblasts. More realistic experimental models should further include 3D tumor-driven angiogenesis and cell spreading, as well as additional ECM-type specific entities interacting with cells (e.g. basement membrane proteins, such as laminin and collagen IV).

## 6 Future directions and concluding remarks

A profound body of evidence indicates that aberrations in the mechanics of the ECM significantly contribute to tumor progression and metastasis. Therefore, there is an increasing need for new techniques to resolve spatiotemporal changes in ECM mechanics and its underpinning biology. However, inherent limitations associated with optical and mechanical tools impose challenges toward capturing high resolution spatiotemporal changes of ECM mechanics in vivo. In vitro methodologies based on the combination of 3D cultures with microfluidic techniques are ideal platforms that can realistically and efficiently recapitulate various bio-mechanical elements of the TME at specific progression points while monitoring dynamic cell–cell and cell–ECM interactions with high resolution.

Since the ECM plays such a prominent role in cancer progression, modulating ECM mechanics offers the potential for new approaches to cancer therapy. New methods are being actively pursued in several laboratories (see, e.g., [129]) to limit the spread of tumor by the use of drugs that alter TME mechanical properties or their spatial gradients. Related studies are addressing the underlying mechanisms that give rise to matrix remodeling. Further work is needed, however, before we can fully characterize the mechanical complexity of the TME, understand the processes that contribute to it, and finally, how it might be regulated for therapeutic benefit.
